# ppGpp signaling plays a critical role in virulence of *Acinetobacter baumannii*

**DOI:** 10.1080/21505594.2021.1961660

**Published:** 2021-08-10

**Authors:** Kyeongmin Kim, Maidul Islam, Hye-won Jung, Daejin Lim, Kwangsoo Kim, Sung-Gwon Lee, Chungoo Park, Je Chul Lee, Minsang Shin

**Affiliations:** a Department of Microbiology, School of Medicine, Kyungpook National University, Jung-gu, Daegu, South Korea; b Department of Microbiology, Chonnam National University Medical School, GwangjuSouth Korea; c School of Biological Sciences and Technology, Chonnam National University, GwangjuSouth Korea

**Keywords:** *Acinetobacter baumannii*, ppGpp, transcriptome, biofilm, virulence

## Abstract

*Acinetobacter baumannii*, a major nosocomial pathogen, survives in diverse hospital environments, and its multidrug resistance is a major concern. The ppGpp-dependent stringent response mediates the reprogramming of genes with diverse functions in several bacteria. We investigated whether ppGpp is involved in *A. baumannii’s* pathogenesis by examining biofilm formation, surface motility, adhesion, invasion, and mouse infection studies. Transcriptome analysis of early stationary phase cultures revealed 498 differentially-expressed genes (≥ 2-fold change) in a ppGpp-deficient *A. baumannii* strain; 220 and 278 genes were up and downregulated, respectively. C*su* operon expression, important in pilus biosynthesis during early biofilm formation, was significantly reduced in the ppGpp-deficient strain. Our findings suggest that ppGpp signaling influences *A. baumannii* biofilm formation, surface motility, adherence, and virulence. We showed the association between ppGpp and pathogenicity in *A. baumannii* for the first time; ppGpp may be a novel antivirulence target in *A. baumannii.*

## Introduction

*Acinetobacter baumannii* is a Gram-negative, strictly aerobic coccobacillus renowned for persistence in diverse environmental conditions [1]. The environmental persistence of *A. baumannii* depends on desiccation resistance, biofilm formation, motility, and resistance to major antimicrobials [2–4]. *A. baumannii* also cause ventilator-associated pneumonia, bloodstream, surgical wounds, and urinary tract infections in humans [5,6]. Moreover, *A. baumannii* spreads in nonhospital communities and may be life-threatening due to treatment failures [7]. Several virulence factors of *A. baumannii* pathogenicity have been identified, including the outer membrane protein A [8,9], biofilm formation and adhesion on biotic and abiotic surfaces [10–12], the uptake of iron, zinc, and other micronutrients [13–15], motility [16,17], production of cytotoxins, and evasion of the innate immune response [18,19].

Biofilms are an assemblage of microbial cells and associate irreversibly with certain surfaces surrounded by a polysaccharide matrix [20]. Biofilm formation is a crucial step in the life cycle of many bacterial species implicated in infection and responsible for approximately 65% of human bacterial disease [21,22]. *A. baumannii* forms biofilms that allow continuous growth under challenging conditions and, through strong adherence to surfaces or tissues, prevent detachment by water or blood flow [23]. *A. baumannii* pili biosynthesis helps form biofilms assembled via the CsuA/BABCDE chaperone-usher secretion system [24]. Pili produced with CsuA/BABCDE play vital roles in the preliminary steps of biofilm formation that allow planktonic cells to attach and start a microcolony formation [25]. A two-component regulatory system controls the expression of the *csuA/BABCDE* operon, and it comprises a sensor kinase that is encoded by *bfmS* with a response regulator that is encoded by *bfmR* [26]. Bacteria form a monolayer and produce an extracellular polymeric matrix after pilus attachment to an abiotic surface, followed by the complete development of the biofilm structure.

Bacterial adherence to epithelial cells and tissues is vital for colonization and host infection [27]. Bacterial viability and cultivability reduce during nutrient starvation, impairs adhesion and invasion potential [28]. In addition to biofilm formation, surface motility is a widespread trait among *A. baumannii* strains and is a major factor for colonization in tissue and hospital environments [29].

The universal signaling alarmones, guanosine tetraphosphate (ppGpp) and guanosine pentaphosphate (pppGpp) (known as [p]ppGpp when combined), are produced under nutrient deprivation [30]. ppGpp is a derivative of hyperphosphorylated guanosine [31,32]. The *RelA* protein present in *Betaproteobacteria* and *Gammaproteobacteria* is responsible for accumulating ppGpp during amino acid starvation. *SpoT* is a homolog of *RelA* and regulates ppGpp accumulation. The *SpoT* protein has synthetic and hydrolytic activities for ppGpp, although its synthetase activity is weak compared with *RelA* [33]. ppGpp is a virulence factor in numerous Gram-negative bacteria, including *Vibrio cholera, Salmonella enterica, Escherichia coli*, and *Pseudomonas aeruginosa* [34–37]. The role of ppGpp in biofilm formation and adhesion in several bacteria has also been described [30,38–41]. According to a recent study, *A. baumannii* ppGpp was involved in motility and virulence in *Galleria mellonella* [42]. In this study compared the transcriptomes, biofilm formation, adherence, and pathogenic properties of wild-type *A. baumannii* and a ppGpp-deficient strain. Overall, our data provide evidence that ppGpp directly or indirectly affects biofilm formation, differential gene expression, and adherence in *A. baumannii*. Targeting ppGpp may be a new therapeutic approach for *A. baumannii* infection.

## Results

### *RNA transcriptome of ppGpp-deficient* A. baumannii

**Figure 1.**
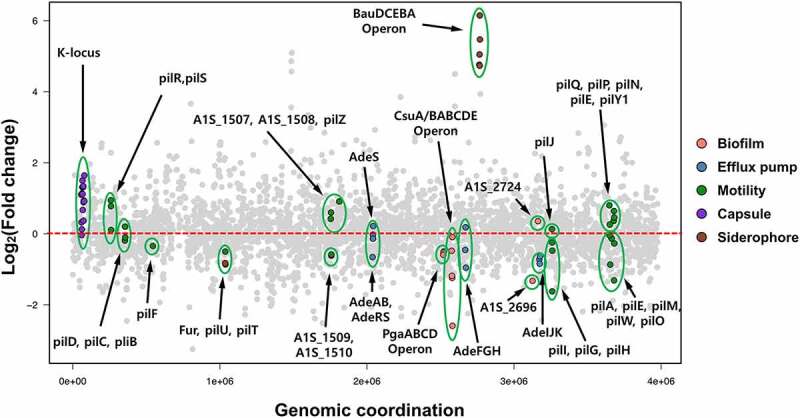
**Overview of differential expression of transcriptomes in the ppGpp-deficient and ATCC 17,978 wild-type strains**. Comparative transcriptome analysis of the ppGpp-deficient strain relative to *Acinetobacter baumannii* ATCC 17,978 was performed using the Illumina platform. Differential expression levels of all predicted open reading frames of the ATCC 17,978 genome are presented as dots. Differentially-expressed genes sorted according to the locus tag on the X- and Y-axis indicate fold change in the ppGpp-deficient strain compared with the wild-type. Upregulated and downregulated genes are separated using a red dashed line. Genes related to biofilm production, efflux pumps, motility, capsule formation, and siderophore production are represented as orange, blue, green, purple, and brown dots, respectively

We previously reported that the ppGpp alarmone regulates efflux pump-related gene expression in *A. baumannii* [43]. Depletion of ppGpp production alters several biological functions of *A. baumannii* as outlined above. We further characterized the effects of ppGpp deficiency in *A. baumannii* by sequencing RNA extracted from early stationary phase samples. We determined the changes in differentially-expressed transcripts by comparing the wild-type and ppGpp-deficient strains (Figure 1). A total of 498 genes were differentially expressed, including 220 genes that were upregulated at least 2-fold and 278 genes that were downregulated at least 2-fold in the ppGpp-deficient strain (Figure 1). Among the differentially-expressed genes, an iron acquisition siderophore gene (A1S_2387) was most upregulated (+ 42.11 fold change), and A1S_2347 that encodes a hypothetical protein was the most downregulated (− 11.27 fold change) in the mutant strain. Genes that show at least 2-fold changes in expression in the ppGpp-deficient strain compared with the wild type are presented in Supplementary Table 1. The differentially-expressed genes were categorized into functional groups based on the Kyoto Encyclopedia of Genes and Genomes pathway. Five gene groups are highlighted in Figure 1, including three groups that were downregulated in the ppGpp-deficient strain and responsible for biofilm production, act as multidrug efflux pumps, or are involved in motility. Expression of genes in the *csu* operon (*A1S_2213-2218*) and *pgaABCD* chaperone-usher system genes (*A1S_1509, A1S_1510*) that synthesize cell-associated poly-beta-(1-6)-N-acetylglucosamine (PNAG) decreased in the ppGpp-deficient strain . *A. baumannii* pellicle and biofilm formation are controlled by the *csu* operon and proteins encrypted by the chaperone-usher system [44,45]. The multidrug efflux pump-related genes, *adeIJK, adeFGH, and adeAB*, were also downregulated in the ppGpp-deficient background [43]. The expression of various pili-related genes responsible for surface motility and adherence of *A. baumannii* was also decreased in the absence of ppGpp (Figure 1). Numerous upregulated genes encoded in the ppGpp-deficient strain are involved in siderophore production, capsule formation, and bacterial motility. K-locus genes determine surface polysaccharides and lipopolysaccharides of *A. baumannii*. The expression of capsular K-locus genes increased in the ppGpp-deficient strain. The *bauDCEBA* siderophore-related gene cluster and pili-related genes were also upregulated in ppGpp-deficient conditions. In summary, genes involved in biofilm formation, siderophore production, adherence to and invasion, and surface motility showed significant changes in expression in the ppGpp-deficient strain of *A. baumannii* than the wild-type strain. We also performed qRT-PCR of several genes using the same RNA sample related to siderophore production (A1S_1921, A1S_2081, A1S_2389), capsule production (A1S_0049, A1S_0050, A1S_0061, A1S_0062) and motility related genes (A1S_0414, A1S_2182, A1S_2813, A1S_2814, A1S_2815) to validate transcriptome data (Supplementary Figure 1). Our qRT-PCR results corroborate transcriptome data. Thus, these transcriptome data are consistent with the impairments in biofilm formation, surface motility, and adherence in the ppGpp-deficient mutant as described in the preceding section.

### *ppGpp modulates biofilm formation and surface motility of* A. baumannii

**Figure 2.**
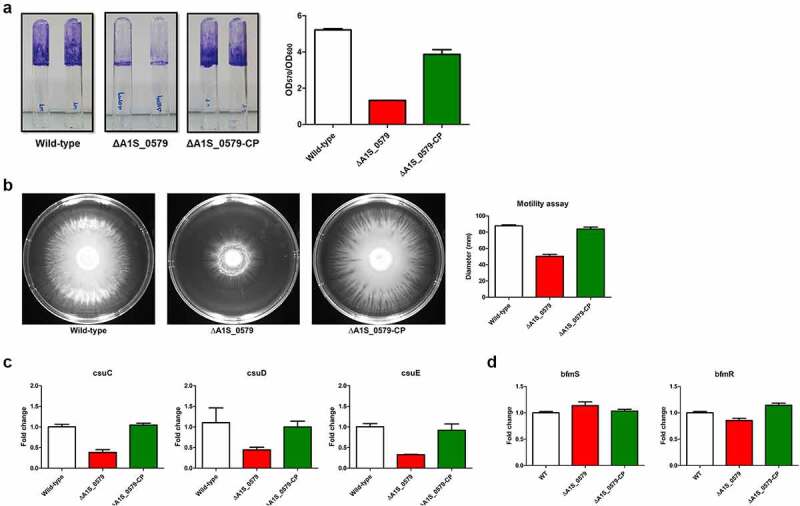
**Effects of ppGpp on biofilm formation and surface motility of *Acinetobacter baumannii* ATCC 17,978 wild-type, ppGpp-deficient, and CP strains**. (a) Biofilm formation on an abiotic (glass) surface. *A. baumannii* ATCC 17,978 wild-type, ppGpp-deficient, and CP strains were cultured in 5 mL polystyrene tubes at 30°C for 24 h in a Luria–Bertani (LB) medium without salt. Biofilms on the tubes were stained with crystal violet (0.1%). Biofilm mass (OD_570_) was normalized by growth levels (OD_600_) to compensate for differences in growth rates. The data are presented as the mean ± standard deviation (SD) of three independent experiments (b) *A. baumannii* strains were grown overnight, and after adjusting to the same optical density, 2 µL of samples were dropped on the surface of modified LB agar (0.3% Eiken agar) plates. LB agar plates were incubated at 37°C for 12 h. Areas of migration were photographed. The diameter of migration (mm) is shown for each strain. The data are presented as the mean ± SD of three independent experiments. (c) RT-qPCR assessed the expression of pili-related *csu* operon (csuC, csuD, and csuE) genes. (d) RT-qPCR assessed the expression of two component system (bfmS, bfmR) related genes. The wild-type value was set to 1, and mutant strains were calculated correspondingly. Error bars represent standard deviations

Biofilm formation is a major factor in the virulence of this pathogen [10]. Biofilm formation assays were performed using a ppGpp-deficient strain to assess whether the alarmone affected *A. baumannii’s* virulence through biofilm production. Biofilm formed by the ppGpp-deficient strain was reduced by approximately 3-fold compared with the parental strain (Figure 2(a)). A strain in which the ppGpp deficiency was complemented (CP strain) yielded less biofilm than the wild-type but produced more biofilm than the ppGpp-deficient strain. We also examined different *A. baumannii* strains’ surface-associated motility with eliciting the effect of ppGpp mutation by measuring colony diameters after 12 h of growth on modified Luria–Bertani (LB) agar plates (Figure 2(b)). The migration ability of the ppGpp-deficient strain from the inoculation area was significantly reduced compared with that of the wild-type strain (20 and 78 mm in the ppGpp-deficient mutant and wild-type, respectively). The impaired motility of the ppGpp mutant strain was recovered in the CP strain (Figure 2(b)). These findings indicate that ppGpp is involved in *A. baumannii’s* biofilm production and surface motility migration.

Biofilm formation in *A. baumannii* is influenced by many genes, including *ompA, csuA/BABCDE, abaI*, and *pgaABCD*. Quantitative PCR (RT-qPCR) was performed to examine the role of ppGpp in the expression of the *csu* operon in the wild-type, ppGpp-deficient, and CP strains. The expression level of *csuC, csuD*, and *csuE* genes was reduced 2.6-fold, 2.5-fold, and 3.1-fold, respectively, in the ppGpp-deficient strain compared with the wild-type strain (Figure 2(c)). The expression levels returned to normal in the CP strain. Also, RT-qPCR was performed for the *bfmS/R* two-component system, a *csu* operon upper regulator. However, there were no notable expression differences of this locus in the wild-type, ppGpp-deficient, and CP strains (Figure 2(d)). These findings confirmed that ppGpp modulates the expression of the *csu* operon, which influences biofilm formation in *A. baumannii*.

### Effects of ppGpp on adherence to and invasion of epithelial cells

**Figure 3.**
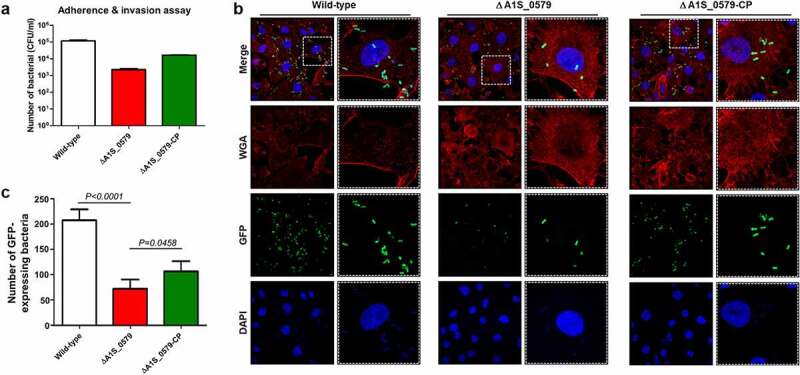
**Effect of ppGpp on the adherence and invasion of *Acinetobacter baumannii* to A549 cells**. (a) *A. baumannii* ATCC 17,978 wild-type, ppGpp-deficient, and CP strains were used to infect A549 human epithelial cells. Data represent the numbers of bacteria bound to and invading A549 cells by colony-forming units on Luria–Bertani agar plates containing trimethoprim. The data are presented as the mean ± standard deviation (SD) of three independent experiments (b) Fluorescent confocal microscopy of A549 cells during infection with *A. baumannii* strains. *A. baumannii* harbored a GFP-expressing plasmid. The cell membrane was stained with wheat germ agglutinin, and the nucleus was stained with DAPI. (c) Quantification of the number of GFP-expressing bacteria that adhered to A549 cells. The data are presented as the mean ± SD

We investigated whether ppGpp is associated with adhesion and invasion of lung epithelial cells, as adhesion is the initial step in colonization and establishment of infection. A549 adenocarcinomic human alveolar basal epithelial cells were inoculated with the wild-type, ppGpp-deficient, and CP strains of *A. baumannii* at multiplicities of infection of 100 for 2 h. Adherence and invasion of A549 cells was evaluated by determining colony-forming units (CFU). Also, the number of adhered bacteria was reduced significantly in the ppGpp-deficient strain relative to the wild-type strain (Figure 3(a)). However, this reduction in the ppGpp-deficient strain partially recovered in the CP strain. The interactions of the three strains with A549 epithelial cells were further studied using plasmid pWH1266, in which the gene for GFP was introduced. This plasmid replicates in *A. baumannii* and allows the observation by confocal microscopy. A significant reduction in adherent bacteria was noted in the ppGpp-deficient strain (Figure 3(b)). Adhesion of wild-type *A. baumannii* to A549 cells was approximately 4-fold higher than by the ppGpp-deficient strain (Figure 3(c)). This adherence reduction in the ppGpp-deficient strain partially recovered in the CP strain. These findings suggest that ppGpp is an essential determinant for adherence and invasion of host epithelial cells by *A. baumannii*.

### *ppGpp regulates* A. baumannii’s *pathogenesis* in vivo

**Figure 4.**
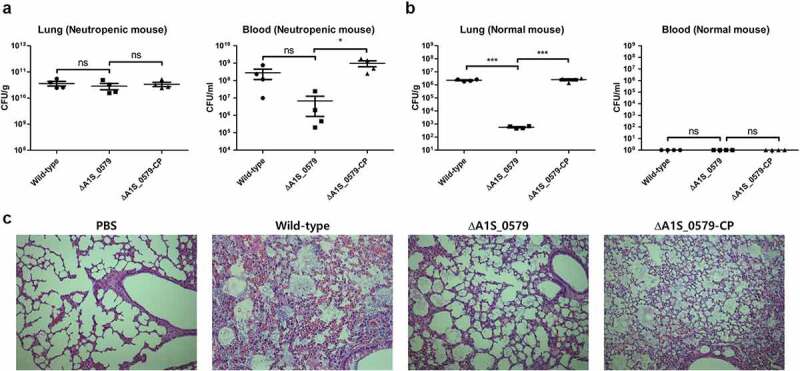
**Contribution of ppGpp to the pathogenesis of *Acinetobacter baumannii.****A. baumannii* strains were injected into neutropenic mice, with phosphate-buffered saline used as a control. Lung and blood samples were collected 24 h after bacterial infection. (a) Colony-forming units (CFU) in lungs and blood of mice. (b) CFU in the lungs of and blood normal mice. The data are presented as the mean ± SD of four determinations of both blood and lung samples. (c) Hematoxylin and eosin staining of lung tissue

The preceding experiments provide a new understanding of ppGpp’s role in the invasion of cell lines by *A. baumannii*. A neutropenic murine pneumonia model was used to observe the role of ppGpp in *A. baumannii’s* pathogenesis *in vivo*. The mice were injected separately with 2 × 10^8^ CFUs of the wild-type, ppGpp-deficient, and CP strains through an intratracheal injection. Mice were euthanized 24 h after infection, and the number of bacteria in the lung and blood samples was enumerated on agar plates, including trimethoprim, by serial dilution. Total bacterial numbers in the lung samples were almost identical to the wild-type, ppGpp-deficient, and CP strains (Figure 4(a)). In contrast, the bacterial number in blood samples was reduced in mice infected with the ppGpp-deficient strain than in the wild-type (Figure 4(a)). Total bacterial counts in mice infected with the wild-type and CP strains were almost similar. These results indicate that ppGpp controls the bloodstream dissemination of *A. baumannii* in mice. In contrast to neutropenic mice, the bacterial burden in the lung samples of normal BALB/C mice was also significantly lower with the ppGpp-deficient strain than wild-type and CP strains (Figure 4(b)). In case of blood samples from normal BALB/C mice, the number of bacteria for all strains was not countable because no bacterial colony was detected in agar plates. We also performed hematoxylin and eosin staining to observe the lung pathology of infected mice (Figure 4(c)). Lung samples from control mice showed normal histological characteristics, whereas the lungs of mice infected with either wild-type or CP strains of *A. baumannii* exhibited inflammation. In contrast, the lungs of mice infected with the ppGpp-deficient strain showed histological characteristics similar to the control. These findings collectively suggest that ppGpp in *A. baumannii* is associated with pathological lung changes and is implicated in disseminating the bacteria into the bloodstream of neutropenic mice.

## Discussion

Bacteria that infect host cells produce adaptive responses to adjust to the novel host environment. The signaling molecule, ppGpp, is involved in several biological processes in bacteria, including antibiotic resistance, biofilm formation, and virulence [46,47]. We recently showed that *A1S_0579* gene is responsible for ppGpp production in *A. baumannii* 17,978 and has a significant role in antibiotic susceptibility [43]. This study focused on the impact of ppGpp deficiency on gene expression profiles, biofilm formation and surface motility, adherence and invasion of epithelial cells, and the *in vivo* virulence of *A. baumannii* in a mouse model.

The secondary messengers, c-di-GMP, cAMP, and ppGpp, are well-known signaling molecules that factor in regulating bacterial biofilm formation and virulence [48–50]. Mutants deficient in ppGpp production tend to exhibit poor biofilm formation properties [51,52]. Thus, our results demonstrated that ppGpp depletion decreased biofilm formation approximately 3-fold in *A. baumannii* and agree with previous observations with other bacteria [51]. However, biofilm formation is enhanced by reduced levels of ppGpp sometimes, implying that the alarmone negatively regulates biofilm formation in genera, such as *Actinobacillus pleuropneumoniae* and *Burkholderia pseudomallei* [39,53]. The reason for these contrasting effects of ppGpp on biofilm production in different bacteria requires further analysis. We found that the key to explaining the effect of ppGpp on biofilm formation by *A. baumannii* potentially relates to pilus production. The *csu* operon is necessary for pilus biosynthesis during early biofilm formation. The expression of genes in the operon decreased in the ppGpp-deficient strain, and defects in biofilm formation accompanied this downregulation. The two-component regulator bfmS/R control biofilm formation and morphology in *A. baumannii* 19,606 [25]. Our qPCR results showed that there was no significant difference in expression of both genes between ppGpp mutant and wild-type although biofilm formation was decreased in ppGpp mutant strain. ppGpp may directly control pilus biosynthesis and biofilm formation without affecting bfmS/R expression. Moreover, our results demonstrated that ppGpp affects biofilm formation and regulates surface motility (Figure 2). Thus, we observed that the surface motility of the ppGpp-deficient strain was impaired compared with the wild-type strain. This result agrees with a previous study showing that a hybrid two-component regulator is involved in biofilm production and motility in *A. baumannii* 17,978 [54]. However, in contrast to our result, another recent study showed high motility in ppGpp-deficient strains by increasing quorum sensing and Acinetin-505 production controlled by LysR-type transcription regulator in clinical *A. baumannii* AB5075 [42]. In our RNA transcriptome results of *A. baumannii* ATCC 17,978, the expression of LysR-type transcription factors in ppGpp deficiency strains has not increased, so we did not investigate this issue. The gene expression profile of the ppGpp mutant strain was analyzed to identify differentially-expressed genes implicated in reducing biofilm formation and motility. These transcriptome data revealed that genes in the *csu* operon and genes related to bacterial motility were downregulated in the ppGpp-defective strain. Also, several other virulence-related genes were identified as differentially expressed, including genes involved in adhesion, capsule formation, and siderophore production. Siderophore biosynthesis enhances the virulence of *Staphylococcus aureus* [55]. Moreover, ppGpp depletion alters the gene expression profile and increases the virulence of *Pseudomonas syringe* [56]. Additional studies are necessary to identify the molecular mechanisms by which differentially-expressed genes contribute to virulence in different backgrounds.

Several previous reports showed numerous *A. baumannii* multidrug-resistant clinical isolates that could adhere to epithelial cells [57]. Specific genes, including *csuC, csuD, csuE*, and *nlpE*, were identified as playing roles in adherence by *A. baumannii*. Disruption of these genes abolished adherence properties [25,58]. We used the ppGpp-deficient mutant to examine the relationship between the alarmone and adherence in *A. baumannii*. Our results showed that ppGpp depletion significantly impaired adherence to A549 epithelial cells compared with the wild-type strain. These observations suggest that ppGpp depletion modulate critical factors that regulate adherence and invasion during infection. Bacteria must disseminate through the bloodstream after transmission via the respiratory tract for disease progression. There was a bacterial reduction in the blood of neutropenic mice infected with the ppGpp-deficient strain compared with the wild-type strain in our mouse pneumonia model but no bacterial colony was detected from blood sample of normal BALB/C mice (Figure 4b). Together, these data reveal that affecting ppGpp production may assist innate immune response-mediated cleaning of *A. baumannii* infections (Figure 4).

In conclusion, this study provides a new understanding of ppGpp’s role in biofilm formation, motility, and adherence in *A. baumannii*. This alarmone is important for survival under different environmental conditions and critical virulence during infection. Our transcriptome data with the ppGpp mutant strain also demonstrated several differentially-expressed genes that maintain the virulence of *A. baumannii*. Moreover, this study shows that ppGpp is a prospective target for expanding antivirulence strategies for *A. baumannii*. Further research should be aimed at developing antimicrobial peptides that target ppGpp production to treat *A. baumannii* infection.

## Methods

### Bacterial strains, plasmids, and culture conditions

*A. baumannii* ATCC 17,978, the ppGpp-deficient strain (∆*A1S_0579* strain), and the CP strain were derived from our previous study [43]. The bacterial strains, plasmids, and list of primers used in this study are listed in Supplementary Tables 2 and 3. Bacterial strains were grown in LB broth or LB broth containing 1.5% (w/v) agar at 30°C or 37°C. Tetracycline (15 µg/mL), ampicillin (100 µg/mL), or both were added to the LB media to maintain plasmids in *E. coli*. After plasmid electroporation, *A. baumannii* strains were selected on LB agar plates supplemented with tetracycline (15 µg/mL).

### Cell culture

Type II pneumocyte cell line A549 was obtained from the Korean Cell Line Bank (Seoul, Korea) and grown in RPMI 1640 medium (HyClone, Logan, UT, USA) supplemented with 10% fetal bovine serum (HyClone), penicillin G (100 U/mL), and streptomycin (50 μg/mL) at 37°C with 5% CO_2_. Cells were harvested, and *A baumannii* strains were infected in six-well plates.

### Isolation of bacterial mRNA and RNA sequencing

Overnight bacterial cultures of *A. baumannii* ATCC 17,978 and ppGpp-deficient mutant strains were freshly cultured in LB by dilution (1%) and grown at 37°C until OD_600_ reached 1.00. Following the manufacturer’s instructions, total RNA was extracted using Qiagen RNeasy Mini kits (Qiagen, Hilden, Germany). Total RNA samples were quantified with a NanoDrop 2000 Spectrophotometer (Thermo Fisher Scientific, Seoul, South Korea). Samples were sent as two biological replicates to Macrogen Inc. (Seoul, South Korea), and the mRNA quality control (QC), cDNA library preparation, library QC, template preparation, template QC, and RNA sequencing on HiSeq 4000 platform were performed. RNA-sequencing reads were aligned with the *A. baumanni* strain ATCC 17,978 (GenBank accession no CP000521.1) using Rockhopper. We discarded any sequencing reads with a fold change less than 2 and *q* value more than 0.05.

### Quantitative PCR for the validation of RNA-sequencing data

Total RNA (1 μg) was synthesized with an M-MLV cDNA Synthesis kit (Enzynomics, Daejeon, South Korea) to generate complementary DNA (cDNA). For RT-qPCR, Primer Express v.3.0 (Applied Biosystems) was used to design primer sequences. RT-qPCR was performed using the ABI Step One Plus Real-Time System (Applied Biosystems) and TOPreal q-PCR 2X PreMIX (SYBR Green with high ROX, Enzynomics). The relative transcript levels were normalized to the level of 16S rRNA, and relative expression was determined by the ΔΔCt method. The primers used in RT-qPCR are listed in Supplementary Table 3. Each RT-qPCR experiment was performed at least three times.

### Biofilm formation assay and quantification

Biofilm formation by *A. baumannii* and its derivative strains was measured using a crystal violet staining assay based on previously outlined procedures [59] with some modifications. Briefly, overnight cultures of *A. baumannii* ATCC 17,978, the ppGpp-deficient, and CP strains were adjusted to an OD_600_ = 1.0 in LB media without sodium chloride and were diluted (1/40). Polystyrene tubes (5 mL) were aliquoted with 2 mL of bacterial suspensions and incubated statically for 24 h at 30°C in a dark room. The tubes were washed two times with 2 mL of distilled water (DW) to remove planktonic or loosely adherent cells. The tubes were air-dried, and the inner walls with the biofilms were stained with 2-mL crystal violet (0.1% v/v) for approximately 15 min. The biofilms were stained with crystal violet were solubilized with 2 mL of 95% ethanol for 5 min. Each sample was transferred to a 96-well plate, and turbidity was measured at 570 nm using a microplate reader (Molecular Devices, Sunnyvale, USA). The samples’ turbidity was also measured at 600 nm before staining the biofilm to calculate for growth differences. Biofilm formation was quantified by calculating the ratio of OD_570_/OD_600_, and each biofilm experiment was performed three times. Also, qRT-PCR was performed to identify the expression levels of the biofilm-related genes. We isolated the total yield of RNAs from respective bacterial strains at an early stationary phase using Qiagen RNeasy Mini kits (Qiagen, Hilden, Germany) as described above. cDNA conversion and quantification of gene transcripts were performed using an ABI Step One Plus^M^ Real-Time System (Applied Biosystems) using Power SYBR Green PCR Master Mix (Applied Biosystems). Sequences of the primers employed for RT-qPCR experiments are shown in Supplementary Table 3. Relative transcript levels were calculated comparing16S rRNA gene C_T_ values and plotted as ∆∆CT. Each experiment was performed at least three times.

### Surface motility assays

Surface-associated motility of *A. baumannii* strains was evaluated following the protocol described in (15). Modified LB agar plates were made using tryptone (10 g/L), yeast extract (5 g/L), and agar (3 g/L; Eiken Chemical). For the motility assay, bacterial strains were grown overnight, and then fresh cultures were made by diluting with fresh LB broth to an OD_600_ = 1.0. Then, 2 µL of bacterial culture was inoculated onto the centers of agar plates. The plates were incubated at 37°C for 12 h and images were taken using a digital imaging system.

### Adherence and invasion assay

The adherence of *A. baumannii* strains to A549 cells was determined as previously described [12]. Briefly, A549 cells were seeded in six-well cell culture dishes at a density of 2 × 10^5^ cells/well. *A. baumannii* strains were cultured up to an OD_600_ = 1.0. *A. baumannii* strains were used to infect cells at multiplicities of infection of 100. After infection with bacteria, cells were incubated at 37°C in 5% CO_2_ for 2 h. The cells were washed five times with PBS and lysed with Triton X-100 (0.1%) at 37°C for 20 min. Lysate samples were serially diluted 10-fold and plated onto LB agar. CFUs were calculated after incubation for 24 h at 37°C. Adherence assays were performed in triplicate.

### *Construction of GFP-tagged* A. baumannii *and confocal microscopy*

To construct *A. baumannii* that expressed GFP, first, we constructed a plasmid with GFP expression and then transferred that plasmid into *A. baumannii*. Briefly, the sfGFP gene was PCR-amplified from the pMR-059 (PT3-sfGFP) plasmid, in which the gene for GFP is under the control of the *ompA* promoter. The PCR-amplified product and pWH1266 vector was ligated by a Gibson assembly reaction to generate the pMI66 plasmid (Supplementary Table 2). Finally, pMI66 was electro transformed into *A. baumannii* to allow for GFP expression in this host (data not shown). Bacterial adherence to A549 cells was investigated by confocal microscopy. A549 cells were grown and infected as described above to visualize the adhesion of *A. baumannii* that expressed GFP. Infected cells were then fixed with paraformaldehyde (4%) and permeabilized with 0.05%. Cells were stained with the Alexa Fluor 594 conjugate of wheat germ agglutinin (Invitrogen/Thermo Fisher Scientific). Cell nuclei were stained with ProLong Gold antifade reagent with DAPI (Invitrogen/Thermo Fisher Scientific). The adherence to and invasion of *A. baumannii* strains were observed using a Zeiss confocal microscope LSM 510 (Zeiss Laboratories), and 488 nm was used to excite GFP and detected at a 515–540 nm bandpass filter. The number of adherent bacteria and the total number of bacteria that interact with cells was counted using a set of 200 cells. Images were acquired, colored, and merged.

### *Systemic infection of mice* in vivo

Eight-week-old female BALB/c mice were maintained under conventional conditions. Neutropenic mice were induced by injecting cyclophosphamide (150 mg/kg) before bacterial infection (days 4 and 1) via intraperitoneal injection [60]. Neutropenic mice were anesthetized with avertin (Sigma Aldrich) via intraperitoneal injection. *A. baumannii* strains (2 × 10^8^ CFU) were injected into neutropenic mice via the intratracheal route. Mice were euthanized and sacrificed 24 h after bacterial infection to obtain blood samples and lung tissue. The right lung tissue of each mouse was weighed and homogenized using Beads crusher µT-12 (TAITEC) twice at 3200 r/min for 60 sec in 500 μL of PBS. The blood and homogenized lung samples were serially diluted 10-fold and plated onto LB agar. CFUs were calculated after incubation for 24 h at 37°C, and the same protocol was followed for normal BALB/C mice. Hematoxylin and eosin stain was used for staining left lung tissues. All experimental animal procedures were approved by the Animal Care Committee of Kyungpook National University, South Korea (approval number: KNU-2019-178).

### Statistical analysis

All data are presented as the mean ± standard deviation of two or three separate experiments. Student’s *t*-test was used to determine the statistical significance of data. A *p*-value less than 0.05 was considered significant.

## Supplementary Material

Supplemental Material

## Date availability

The transcriptomic data discussed in this publication have been deposited in the NCBI’s gene expression omnibus database (http://www.ncbi.nlm.nih.gov/geo/) and can be accessed using the accession number GSE173627.
