# Supplementary material for: ppGpp signaling plays a critical role in virulence of Acinetobacter baumannii
Source: Virulence. 2021 Aug 10;12(1):2122–32. doi: 10.1080/21505594.2021.1961660 (PMC8366539; doi:10.1080/21505594.2021.1961660)
Supplement: Supplemental Material [file KVIR_A_1961660_SM8364.zip › downloadFromZipFile.pdf]

Fig. 1S

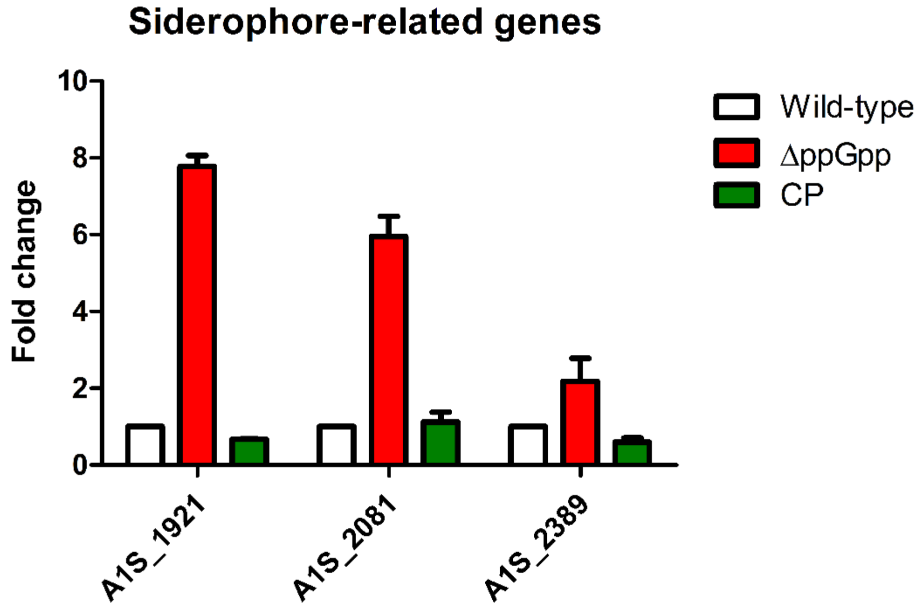

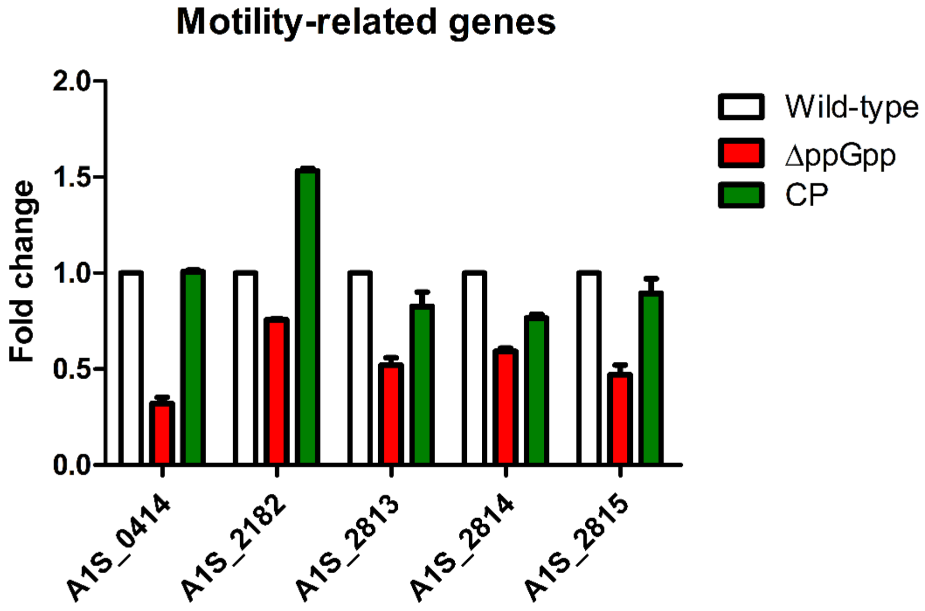

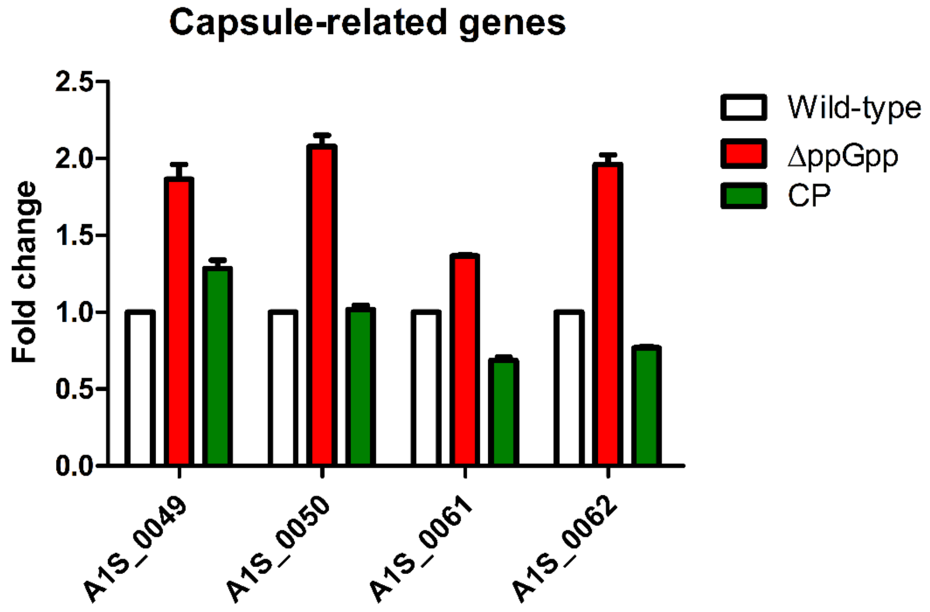

Figure 1S. qRT-PCR validation of transcriptome data.
qRT-PCR analysis of selected differentially expressed genes categorized as different functional groups such as siderophore production (A1S_1921, A1S_2081, A1S_2389), capsule production (A1S_0049, A1S_0050, A1S_0061, A1S_0062) and motility related genes (A1S_0414, A1S_2182, A1S_2813, A1S_2814, A1S_2815) The data represent mean ± standard deviation from three biological replicates.

Table 1S. Differential upregulated gene expression of ΔA1S_1874 mutant versus *A. baumannii* 17978 WT.

Locus_Tag	Gene Product	Fold change (ΔA1S_0579/WT)
A1S_2387	BauE	**42.112641**
A1S_2389	ferric acinetobactin transport system permease protein	**32.438932**
A1S_2388	ferric acinetobactin transport system permease protein	**30.297742**
A1S_2386	ferric acinetobactin binding protein	**25.576735**
A1S_2385	ferric acinetobactin receptor	**21.011321**
A1S_2390	acinetobactin biosynthesis protein	**10.238269**
A1S_1778	methylenetetrahydrofolate reductase	**6.763743**
A1S_1234	tRNA-Gly	**6.576237**
A1S_2271	rtcb protein	**5.272702**
A1S_1647	siderophore biosynthesis protein	**5.015678**
A1S_0980	ferric enterobactin receptor precursor	**4.943418**
A1S_3019	tRNA-Arg	**4.932390**
A1S_1921	ferrichrome-iron receptor	**4.858394**
A1S_2081	TonB-dependent siderophore receptor	**4.847805**
A1S_1700	acetoin:26-dichlorophenolindophenol oxidoreductase subunit beta	**4.701480**
A1S_0332	tRNA-Leu	**4.678324**
A1S_1344	thiolase	**4.499279**
A1S_0294	like chaperone Hsp90	**4.213380**
A1S_0474	ferric siderophore receptor protein	**4.209920**
A1S_1657	siderophore biosynthesis protein	**4.156997**
A1S_1467	glutamate symport transmembrane protein	**4.108950**
A1S_1649	RND efflux transporter	**4.044284**
A1S_2450	pyruvate decarboxylase	**4.001548**
A1S_2045	ferredoxin-dependent glutamate synthase	**3.917137**
A1S_1665	hypothetical protein	**3.899239**
A1S_1796	aldehyde dehydrogenase	**3.894615**
A1S_3253	signal peptide	**3.810641**
A1S_3450	uracil transport protein	**3.808317**
A1S_3443	chaperone protein DnaJ	**3.664752**
A1S_0700	methyltransferase	**3.641994**
A1S_1345	hypothetical protein	**3.640438**
A1S_1343	protein PaaC	**3.609599**
A1S_0673	transposase	**3.471913**
A1S_1699	acetoin:26-dichlorophenolindophenol oxidoreductase subunit alpha	**3.446016**
A1S_1648	lysine/ornithine N-monooxygenase	**3.385548**
A1S_1466	glutaminase-asparaginase	**3.378337**
A1S_0628	transposase	**3.367099**
A1S_2593	group A colicins tolerance protein	**3.296441**
A1S_1342	enoyl-CoA hydratase	**3.247345**

Locus_Tag	Gene Product	Fold change (ΔA1S_0579/WT)
A1S_2077	outer membrane porin receptor for Fe(III)-coprogen, Fe(III)-ferrioxamine B and Fe(III)-rhodotrulic acid uptake (fhuE)	**3.226302**
A1S_3451	uracil transport protein	**3.166606**
A1S_2438	tRNA (5-methyl aminomethyl-2-thiouridylate)-methyltransferase	**3.158940**
A1S_1655	ferric siderophore receptor protein	**3.149943**
A1S_1650	hypothetical protein	**3.107227**
A1S_0387	flavoprotein	**3.101132**
A1S_1063	TonB-dependent siderophore receptor	**2.999617**
A1S_0981	ferric enterobactin receptor precursor	**2.987536**
A1S_1652	hypothetical protein	**2.969371**
A1S_2508	aspartate aminotransferase A	**2.962740**
A1S_2668	phosphoenolpyruvate carboxykinase	**2.956275**
A1S_2606	phosphoribosylglycinamide formyltransferase 1	**2.947630**
A1S_0645	hypothetical protein	**2.936761**
A1S_0886	deoxyuridine 5'-triphosphate nucleotidohydrolase	**2.877211**
A1S_1703	dihydrolipoamide dehydrogenase	**2.873312**
A1S_2515	tRNA hydroxylase	**2.811068**
A1S_3227	RNA binding protein	**2.798734**
A1S_2239	tRNA-Leu	**2.795496**
A1S_2592	group A colicins tolerance protein	**2.791257**
A1S_2080	siderophore receptor	**2.774395**
A1S_1975	uridylate kinase	**2.770099**
A1S_2382	BasD	**2.735706**
A1S_2380	acinetobactin biosynthesis protein	**2.705407**
A1S_1191	aspartate carbamoyltransferase non-catalytic subunit	**2.702202**
A1S_2596	fructose-1,6-bisphosphatase	**2.679072**
A1S_2452	NAD-dependent aldehyde dehydrogenases	**2.678739**
A1S_2793	amino-acid transport protein	**2.674741**
A1S_2674	hypothetical protein	**2.668766**
A1S_0854	NAD-dependent succinate aldehyde dehydrogenases	**2.660604**
A1S_1382	transcriptional regulator	**2.648110**
A1S_0827	peptidyl-tRNA hydrolase	**2.642894**
A1S_0032	signal peptide	**2.639485**
A1S_0007	transport protein	**2.638229**
A1S_1976	30S ribosomal protein S12 methylthiotransferase	**2.636905**
A1S_0317	fusaric acid resistance protein	**2.628336**
A1S_1196	tRNA (guanine-N(7)-)-methyltransferase	**2.593436**
A1S_0888	acetylglutamate kinase	**2.581959**
A1S_2251	amidophosphoribosyltransferase	**2.578344**
A1S_0885	ATP-dependent DNA helicase	**2.576645**
A1S_2026	hypothetical protein	**2.565964**
A1S_3174	regulatory or redox component complexing with Bfr in iron storage and mobility (BFD)	**2.565042**

Locus_Tag	Gene Product	Fold change (ΔA1S_0579/WT)
A1S_0830	tRNA-Gln	**2.560027**
A1S_0799	DNA ligase	**2.546226**
A1S_2836	transporter	**2.541097**
A1S_1149	hypothetical protein	**2.503069**
A1S_2838	lysine-specific permease	**2.486155**
A1S_2963	phosphoribosylaminoimidazole carboxylase ATPase subunit	**2.483737**
A1S_1341	enoyl-CoA hydratase/carnithine racemase	**2.481287**
A1S_1176	alanyl-tRNA synthetase	**2.477595**
A1S_0973	D-serine dehydratase	**2.473683**
A1S_0852	dioxygenase subunit alpha	**2.467799**
A1S_2070	magnesium-transporting ATPase MgtA	**2.449920**
A1S_0853	succinate-semialdehyde dehydrogenase	**2.443325**
A1S_2516	pyridoxine 5'-phosphate synthase	**2.439954**
A1S_3364	VGR-like protein	**2.429907**
A1S_3228	RNA binding protein	**2.426166**
A1S_2298	SMR family efflux pump	**2.418187**
A1S_1694	chorismate synthase	**2.416290**
A1S_0534	NADH-dependent enoyl-ACP reductase	**2.413656**
A1S_1701	dihydrolipoamide acetyltransferase	**2.405335**
A1S_0515	histidine ammonia-lyase protein	**2.402204**
A1S_2968	hypothetical protein	**2.378845**
A1S_0554	peptide chain release factor 3	**2.376392**
A1S_1354	ACP phosphodiesterase	**2.372599**
A1S_1779	MutT/nudix family protein	**2.368730**
A1S_0433	transporter	**2.365063**
A1S_2590	thioesterase	**2.359692**
A1S_1186	ATP-dependent protease Hsp 100	**2.357984**
A1S_3127	signal peptide	**2.357716**
A1S_1600	lysozyme	**2.346099**
A1S_3157	hypothetical protein	**2.346040**
A1S_2244	O-sialoglycoprotein endopeptidase gcp	**2.340967**
A1S_2569	hypothetical protein	**2.337654**
A1S_0969	transketolase	**2.336758**
A1S_2252	colicin V producing membrane protein	**2.330098**
A1S_0952	major facilitator superfamily permease	**2.329171**
A1S_0537	RND efflux transporter	**2.328457**
A1S_2972	guanine deaminase	**2.318651**
A1S_1521	transketolase	**2.310937**
A1S_0664	replication C family protein	**2.308122**
A1S_1190	aspartate carbamoyltransferase catalytic subunit	**2.300497**
A1S_2293	ferredoxin--NADP+ reductase	**2.299134**
A1S_3339	ferric siderophore receptor protein	**2.290383**
A1S_2439	tRNA (5-methyl aminomethyl-2-thiouridylate)-methyltransferase	**2.287660**

Locus_Tag	Gene Product	Fold change (ΔA1S_0579/WT)
A1S_1651	hypothetical protein	**2.280584**
A1S_3211	S-methylmethionine APC transporter	**2.277744**
A1S_0826	peptidyl-tRNA hydrolase	**2.271946**
A1S_0666	TrbL/VirB6 plasmid conjugal transfer protein	**2.268496**
A1S_1348	carbonic anhydrase	**2.267834**
A1S_3130	succinylglutamic semialdehyde dehydrogenase	**2.260380**
A1S_0630	hypothetical protein	**2.255783**
A1S_2656	C32 tRNA thiolase	**2.251718**
A1S_2312	transport protein	**2.248490**
A1S_1797	aldehyde dehydrogenase	**2.248190**
A1S_2188	hypothetical protein	**2.247788**
A1S_2384	BasC	**2.241621**
A1S_0984	carbonic anhydrase	**2.239436**
A1S_1543	phosphoglycerate kinase	**2.238074**
A1S_2115	methyl-directed mismatch repair enzyme	**2.235712**
A1S_0971	B12-dependent methionine synthase	**2.235318**
A1S_3238	1-(5-phosphoribosyl)-5-[(5-phosphoribosylamino) methylideneamino] imidazole-4-carboxamide isomerase	**2.234792**
A1S_0325	hypothetical protein	**2.229989**
A1S_1027	signal peptide	**2.229750**
A1S_1751	AdeA membrane fusion protein	**2.228519**
A1S_0481	phosphate acetyltransferase	**2.227716**
A1S_2504	excinuclease ABC subunit B	**2.223392**
A1S_2370	uroporphyrinogen decarboxylase	**2.220478**
A1S_1888	transport protein	**2.217458**
A1S_0936	hypothetical protein	**2.216342**
A1S_2837	ribosomal RNA small subunit methyltransferase C	**2.215070**
A1S_2289	signal peptide	**2.209533**
A1S_2835	GTP-binding elongation factor family protein	**2.204689**
A1S_2966	UDP-N-acetylmuramate--L-alanyl-gamma-D-glutamyl-meso-diaminopimelate ligase	**2.203808**
A1S_2272	hypothetical protein	**2.201026**
A1S_2440	hypothetical protein	**2.199718**
A1S_2846	CysI-like sulfite reductase protein	**2.194721**
A1S_0576	hypothetical protein	**2.192677**
A1S_1059	oligopeptide transport protein	**2.190460**
A1S_2321	hypothetical protein	**2.175566**
A1S_2585	phosphoribosylformylglycinamidine synthase	**2.175326**
A1S_2756	esterase	**2.164899**
A1S_2597	tRNA/rRNA methyltransferase	**2.163275**
A1S_1862	hypothetical protein	**2.159879**
A1S_1890	3-carboxy-cis, cis-muconate cycloisomerase	**2.158948**
A1S_2973	guanine deaminase	**2.158214**

Locus_Tag	Gene Product	Fold change (ΔA1S_0579/WT)
A1S_0516	hypothetical protein	**2.157925**
A1S_1024	endonuclease III	**2.153601**
A1S_3156	hypothetical protein	**2.147736**
A1S_1064	tRNA-Glu	**2.147074**
A1S_1818	MaoC-like dehydratase	**2.146646**
A1S_2441	adenylosuccinate lyase	**2.131663**
A1S_2804	tRNA-Asp	**2.126269**
A1S_0480	fumarate hydratase	**2.122604**
A1S_1526	hypothetical protein	**2.121709**
A1S_0514	hypothetical protein	**2.120005**
A1S_0831	tRNA-Gln	**2.119089**
A1S_2187	hypothetical protein	**2.116442**
A1S_0909	MFS family transporter	**2.108473**
A1S_3093	nucleoside-diphosphate-sugar epimerase	**2.107906**
A1S_2964	phosphoribosylaminoimidazole carboxylase mutase subunit	**2.107179**
A1S_2184	50S ribosomal protein L11 methyltransferase	**2.103190**
A1S_0940	hypothetical protein	**2.099067**
A1S_1142	aspartate kinase	**2.097027**
A1S_2307	serine hydroxymethyltransferase	**2.094207**
A1S_2013	MFS superfamily metabolite transporter	**2.085043**
A1S_1280	transcriptional regulator	**2.082907**
A1S_1083	aromatic amino acid APC transporter	**2.081358**
A1S_2375	ABC transporter	**2.081299**
A1S_1097	hypothetical protein	**2.077202**
A1S_2845	hypothetical protein	**2.076933**
A1S_3284	transcriptional regulator	**2.076332**
A1S_1898	phosphopyruvate hydratase	**2.075505**
A1S_3429	hypothetical protein	**2.073471**
A1S_2501	glyceraldehyde-3-phosphate dehydrogenase	**2.073125**
A1S_0324	tRNA/rRNA methyltransferase	**2.069228**
A1S_0947	vanillate O-demethylase oxygenase subunit (VanA-like)	**2.065956**
A1S_1799	multidrug efflux MFS transporter	**2.062588**
A1S_3254	KUP family potassium transport system low affinity	**2.060357**
A1S_0765	uracil phosphoribosyltransferase	**2.047815**
A1S_1068	argininosuccinate synthetase	**2.046872**
A1S_2503	outer membrane lipoprotein	**2.045111**
A1S_0644	hypothetical protein	**2.043696**
A1S_1481	GTP-binding protein	**2.041902**
A1S_3324	ferric siderophore receptor protein	**2.041575**
A1S_0555	hypothetical protein	**2.035941**
A1S_0523	3-hydroxylacyl-(acyl carrier protein) dehydratase	**2.035492**
A1S_1248	hypothetical protein	**2.035410**

A1S_2182	tRNA uridine 5-carboxymethylaminomethyl modification protein GidA	**2.029001**
A1S_0522	3-oxoacyl-ACP synthase	**2.027813**
A1S_1653	hypothetical protein	**2.025586**
A1S_0066	hypothetical protein	**2.025581**
A1S_2379	histidine decarboxylase	**2.021965**
A1S_1192	aspartate carbamoyltransferase non-catalytic subunit	**2.016894**
A1S_2369	hypothetical protein	**2.016812**
A1S_0170	outer membrane copper receptor (OprC)	**2.014635**
A1S_2286	hypothetical protein	**2.013969**
A1S_2324	methionine aminopeptidase	**2.004492**
A1S_1160	hypothetical protein	**2.004361**
A1S_1558	DNA polymerase III subunit delta'	**2.004358**
A1S_2435	D-ala-D-ala-carboxypeptidase	**2.001566**
A1S_2361	hypothetical protein	**2.000346**

Difference of >2-fold (q value < 0.05).

Table 1S. Differential downregulated gene expression of ppGpp deficient mutant versus *A. baumannii* 17978 WT.

Locus_Tag	Gene Product	Fold change (ΔA1S_0579/WT)
A1S_2347	hypothetical protein	-11.274816
A1S_3350	hypothetical protein	-6.150231
A1S_2512	hypothetical protein	-5.755787
A1S_2078	acetyltransferase	-4.996468
A1S_3363	membrane metalloendopeptidases proteins	-4.989501
A1S_2961	hypothetical protein	-4.745193
A1S_0683	Sigma (54) modulation protein RpoX	-4.478762
A1S_2071	hypothetical protein	-4.471746
A1S_3377	hypothetical protein	-4.329572
A1S_0189	hypothetical protein	-4.300473
A1S_1263	L-2-haloalkanoic acid dehalogenase	-4.182902
A1S_1687	transcriptional regulator	-4.092599
A1S_1933	hypothetical protein	-4.051702
A1S_3362	hypothetical protein	-3.895769
A1S_2859	hemolysin III (HLY-III)	-3.843926
A1S_1935	hypothetical protein	-3.833550
A1S_1498	TetR family transcriptional regulator	-3.745622
A1S_1499	hypothetical protein	-3.734063
A1S_3473	hypothetical protein	-3.647569
A1S_2072	universal stress family protein	-3.646871
A1S_2757	hypothetical protein	-3.637543
A1S_1356	4-hydroxybenzoate 3-monooxygenase	-3.613335
A1S_1490	glutamate/aspartate transport protein	-3.609150
A1S_2886	acyl-CoA dehydrogenase	-3.433944
A1S_2483	hypothetical protein	-3.420615
A1S_2772	quinone family NAD(P)H dehydrogenase	-3.397524
A1S_0077	hypothetical protein	-3.337492
A1S_2841	type 4 fimbrial biogenesis protein FimT	-3.334120
A1S_0236	response regulator	-3.319023
A1S_0682	RNA polymerase factor sigma-54	-3.305352
A1S_0250	adenosine diphosphate sugar pyrophosphatase	-3.302134
A1S_3111	acyl-CoA dehydrogenase	-3.248869
A1S_2061	3-ketoacyl-ACP reductase	-3.236601
A1S_1266	hypothetical protein	-3.236449
A1S_3054	monooxygenase	-3.234427
A1S_2583	hypothetical protein	-3.215479
A1S_0040	oxidoreductase	-3.180965
A1S_1246	universal stress protein	-3.156693
A1S_2209	acetyltransferase	-3.146444
A1S_0422	AraC family transcriptional regulator	-3.129130
A1S_3108	coproporphyrinogen III oxidase	-3.091116

Locus_Tag	Gene Product	Fold change (ΔA1S_0579/WT)
A1S_3212	hypothetical protein	-3.087033
A1S_0719	zinc-binding dehydrogenase	-3.086016
A1S_2042	TetR family transcriptional regulator	-3.084523
A1S_2348	hypothetical protein	-3.083684
A1S_2814	twitching motility protein	-3.060478
A1S_0289	hypothetical protein	-3.059063
A1S_3208	peptide signal	-3.057916
A1S_2210	hypothetical protein	-3.051916
A1S_2758	membrane protease subunit	-3.047291
A1S_2816	hypothetical protein	-3.038270
A1S_0740	phage related protein	-3.033863
A1S_2815	twitching motility protein	-3.022701
A1S_3031	hypothetical protein	-3.010678
A1S_1370	oxidoreductase	-3.002026
A1S_0548	TetR family transcriptional regulator	-2.993423
A1S_3024	hypothetical protein	-2.991764
A1S_0966	transcriptional regulator	-2.991120
A1S_2205	paraquat-inducible protein A	-2.987712
A1S_2493	2-nitropropane dioxygenase	-2.962492
A1S_1267	lactam utilization protein	-2.957234
A1S_1924	cytochrome d terminal oxidase polypeptide subunit I	-2.952394
A1S_1163	hypothetical protein	-2.948174
A1S_1925	cytochrome d terminal oxidase polypeptide subunit II	-2.932744
A1S_2062	acetyl-CoA acetyltransferase	-2.929530
A1S_3188	tRNA-Glu	-2.928237
A1S_0796	GntR family transcriptional regulator	-2.925944
A1S_2863	antioxidant protein	-2.909866
A1S_0794	hypothetical protein	-2.883130
A1S_1680	hypothetical protein	-2.829836
A1S_0249	cyclic 3'5'-adenosine monophosphate phosphodiesterase	-2.821585
A1S_2484	hypothetical protein	-2.807860
A1S_2629	site-specific tyrosine recombinase	-2.802834
A1S_1489	glutathione S-transferase	-2.798549
A1S_2428	ATP-dependent protease	-2.786750
A1S_3283	gamma-aminobutyrate permease	-2.783519
A1S_2690	hypothetical protein	-2.781759
A1S_2905	bifunctional glutamine-synthetase adenylyltransferase/deadenyltransferase	-2.775153
A1S_0783	hypothetical protein	-2.772968
A1S_2771	AraC family transcriptional regulator	-2.761815
A1S_2738	hypothetical protein	-2.757289
A1S_3226	tRNA-Ser	-2.745638

Locus_Tag	Gene Product	Fold change (ΔA1S_0579/WT)
A1S_1443	taurine ATP-binding transport system component	**-2.742870**
A1S_2218	protein CsuA/B	**-2.728143**
A1S_0912	50S ribosomal protein L22	**-2.713633**
A1S_0371	hypothetical protein	**-2.711803**
A1S_1445	taurine dioxygenase	**-2.703911**
A1S_0572	3-hydroxyisobutyrate dehydrogenase or 2-hydroxy-3-oxopropionate reductase	**-2.703268**
A1S_2648	hypothetical protein	**-2.701934**
A1S_2235	regulatory protein LysR:LysR, substrate-binding	**-2.700180**
A1S_0573	enoyl-CoA hydratase	**-2.698080**
A1S_2785	protease	**-2.698060**
A1S_1948	MarR family multidrug resistance pump transcriptional regulator	**-2.678072**
A1S_1776	transcriptional regulator	**-2.673686**
A1S_2994	hypothetical protein	**-2.665724**
A1S_0459	hypothetical protein	**-2.661082**
A1S_1958	transcriptional regulator	**-2.659381**
A1S_2202	aspartate racemase	**-2.658206**
A1S_3474	hypothetical protein	**-2.646210**
A1S_2228	hypothetical protein	**-2.643865**
A1S_2887	acyl-CoA dehydrogenase A	**-2.643434**
A1S_2975	hypothetical protein	**-2.640424**
A1S_1446	hydantoin racemase	**-2.633199**
A1S_0162	TetR/AcrR family transcriptional regulator	**-2.629508**
A1S_0184	hypothetical protein	**-2.613211**
A1S_1071	tRNA-Ser	**-2.611888**
A1S_0034	short chain dehydrogenase	**-2.610758**
A1S_1926	hypothetical protein	**-2.606908**
A1S_2864	hypothetical protein	**-2.605192**
A1S_2906	sensory transduction histidine kinase	**-2.596166**
A1S_3141	partition-related protein	**-2.592595**
A1S_1070	24-dienoyl-CoA reductase	**-2.588074**
A1S_1444	ABC taurine transporter permease subunit	**-2.582187**
A1S_3014	hypothetical protein	**-2.571264**
A1S_0207	hypothetical protein	**-2.570753**
A1S_1399	ArtI protein	**-2.562075**
A1S_0311	acyl-CoA thioesterase II	**-2.550556**
A1S_0449	coniferyl aldehyde dehydrogenase (CALDH)	**-2.535776**
A1S_1195	glutathione S-transferase	**-2.520543**
A1S_0078	hypothetical protein	**-2.519014**
A1S_2225	hypothetical protein	**-2.506645**
A1S_2429	ATP-dependent protease	**-2.502594**
A1S_2685	hypothetical protein	**-2.493456**

Locus_Tag	Gene Product	Fold change (ΔA1S_0579/WT)
A1S_0209	transposase	-2.492930
A1S_2908	integrase	-2.492744
A1S_0312	CDP-diacylglycerol--glycerol-3-phosphate 3-phosphatidyltransferase	-2.487804
A1S_3172	hypothetical protein	-2.484629
A1S_3237	exonuclease	-2.482881
A1S_1377	acrR family transcriptional regulator	-2.479053
A1S_0891	hypothetical protein	-2.474536
A1S_1199	glutathionine S-transferase	-2.472768
A1S_0696	MutT/nudix family protein	-2.471424
A1S_0911	hypothetical protein	-2.467739
A1S_2281	hypothetical protein	-2.466306
A1S_2950	hypothetical protein	-2.461836
A1S_1802	hypothetical protein	-2.461100
A1S_2229	acyl-CoA dehydrogenase-related protein	-2.460802
A1S_1738	transcriptional regulator	-2.449967
A1S_3281	4-aminobutyrate aminotransferase	-2.448970
A1S_3159	lipase chaperone	-2.446929
A1S_0877	threonine efflux system	-2.438046
A1S_3380	nicotinate phosphoribosyltransferase	-2.433020
A1S_3046	oligopeptidase A	-2.429129
A1S_2662	hydrolase	-2.414451
A1S_2928	hypothetical protein	-2.411321
A1S_0865	hypothetical protein	-2.393565
A1S_2911	hypothetical protein	-2.390877
A1S_0219	ammonium transporter	-2.386432
A1S_0320	hypothetical protein	-2.383579
A1S_1643	acyl-CoA dehydrogenase	-2.381444
A1S_1743	oxygenase subunit protein	-2.381335
A1S_2008	DNA repair protein	-2.376719
A1S_3144	hypothetical protein	-2.375412
A1S_3285	hypothetical protein	-2.374146
A1S_2320	AraC family transcriptional regulator	-2.354780
A1S_1318	N-acetyltransferase GCN5	-2.351259
A1S_0261	alginate biosynthesis regulatory protein	-2.347807
A1S_3265	hypothetical protein	-2.347497
A1S_1501	Phage integrase	-2.342277
A1S_3233	hypothetical protein	-2.341209
A1S_2177	proteasome protease	-2.332065
A1S_3037	ribonuclease (Rbn)	-2.321409
A1S_2488	hypothetical protein	-2.313286
A1S_0344	ATP binding site	-2.312397

Locus_Tag	Gene Product	Fold change (ΔA1S_0579/WT)
A1S_1254	hypothetical protein	**-2.311895**
A1S_0813	hypothetical protein	**-2.311140**
A1S_1449	transcriptional regulator	**-2.310783**
A1S_2813	twitching motility protein	**-2.310139**
A1S_1187	CinA-like protein	**-2.305936**
A1S_3262	transcriptional regulator	**-2.304645**
A1S_0414	dinucleoside polyphosphate hydrolase	**-2.302161**
A1S_2776	hypothetical protein	**-2.301492**
A1S_0750	hypothetical protein	**-2.298830**
A1S_1618	hypothetical protein	**-2.296763**
A1S_3180	signal peptide	**-2.292824**
A1S_1855	transcriptional regulator	**-2.292008**
A1S_3036	tryptophan repressor binding protein	**-2.291218**
A1S_0690	protein FilA	**-2.286274**
A1S_0476	ATP-dependent Clp protease proteolytic subunit	**-2.281715**
A1S_0412	catalase	**-2.276387**
A1S_0986	transport protein	**-2.275353**
A1S_0079	hypothetical protein	**-2.273667**
A1S_0491	hypothetical protein	**-2.270833**
A1S_1984	D-amino acid dehydrogenase small subunit	**-2.268172**
A1S_1366	amino acid transporter LysE	**-2.260965**
A1S_0372	protein secretion chaperone	**-2.259945**
A1S_2461	hypothetical protein	**-2.257359**
A1S_1471	AraC family transcriptional regulator	**-2.256894**
A1S_3368	hypothetical protein	**-2.252320**
A1S_1708	beta-lactamase-like protein	**-2.245660**
A1S_0479	signal peptide	**-2.243736**
A1S_1746	transcriptional regulator	**-2.241026**
A1S_1672	hypothetical protein	**-2.239733**
A1S_1755	AdeT	**-2.239319**
A1S_0987	hypothetical protein	**-2.237800**
A1S_1908	phospho-2-dehydro-3-deoxyheptonate aldolase	**-2.237388**
A1S_0621	two-component response regulator	**-2.221523**
A1S_0478	signal peptide	**-2.217432**
A1S_1540	TetR family transcriptional regulator	**-2.207748**
A1S_3309	acetyl-CoA synthetase	**-2.196621**
A1S_1075	D-amino-acid dehydrogenase	**-2.192506**
A1S_1470	glutathione peroxidase	**-2.190629**
A1S_2773	long-chain fatty acid transport protein	**-2.189736**
A1S_2787	hypothetical protein	**-2.189256**
A1S_2032	hypothetical protein	**-2.187885**
A1S_1455	transcriptional regulator	**-2.182792**
A1S_0432	transporter protein	**-2.181960**

A1S_2267	hypothetical protein	**-2.179956**
A1S_2822	hypothetical protein	**-2.178685**
A1S_2151	AraC family transcriptional regulator	**-2.176275**
A1S_2941	copper resistance D	**-2.172200**
A1S_1089	hypothetical protein	**-2.171805**
A1S_2834	large-conductance mechanosensitive channel	**-2.170796**
A1S_2582	AraC family transcriptional regulator	**-2.166392**
A1S_2430	ATP-dependent protease	**-2.158128**
A1S_2770	fatty acid desaturase	**-2.156585**
A1S_0319	Holliday junction resolvase-like protein	**-2.148584**
A1S_3464	Cro-like protein	**-2.143504**
A1S_0394	acyl-CoA dehydrogenase	**-2.142673**
A1S_3278	isochorismatase superfamily hydrolase	**-2.134323**
A1S_0997	hypothetical protein	**-2.133970**
A1S_3051	hypothetical protein	**-2.132845**
A1S_0438	hypothetical protein	**-2.131176**
A1S_0218	nitrogen assimilation regulatory protein P-II 2	**-2.127496**
A1S_0490	hydrolase	**-2.127142**
A1S_1999	molybdopterin biosynthesis protein A	**-2.122888**
A1S_1203	o-methyl transferase	**-2.121324**
A1S_2672	signal peptide	**-2.119941**
A1S_0392	ABC1 protein	**-2.113915**
A1S_0038	transcriptional regulator	**-2.112368**
A1S_0297	hypothetical protein	**-2.106651**
A1S_3109	shikimate 5-dehydrogenase	**-2.106458**
A1S_3030	phosphate starvation-inducible protein (PhoH-like)	**-2.100418**
A1S_3470	regulatory protein LysR	**-2.098399**
A1S_0489	gamma-glutamyl phosphate reductase	**-2.090178**
A1S_2486	hypothetical protein	**-2.089279**
A1S_0779	hypothetical protein	**-2.083540**
A1S_3241	polyketide synthesis monooxygenase	**-2.082679**
A1S_0419	hypothetical protein	**-2.081732**
A1S_2919	hypothetical protein	**-2.081470**
A1S_3195	membrane protein ComM	**-2.080685**
A1S_2762	aromatic amino acid APC transporter	**-2.080410**
A1S_3465	hypothetical protein	**-2.080331**
A1S_3286	inner membrane protein	**-2.079788**
A1S_1863	hypothetical protein	**-2.078547**
A1S_2060	dehydratase	**-2.078224**
A1S_0697	MutT/nudix family protein	**-2.073768**
A1S_0208	hypothetical protein	**-2.072564**
A1S_3411	G3E family GTPase	**-2.070736**
A1S_3038	hypothetical protein	**-2.070505**
A1S_0695	protein FilF	**-2.066689**

A1S_2485	glycosyltransferase	**-2.066361**
A1S_2728	hypothetical protein	**-2.066348**
A1S_1762	hypothetical protein	**-2.064813**
A1S_0306	similar to fatty oxidation complex alpha subunit	**-2.061220**
A1S_2860	MFS family transporter	**-2.061004**
A1S_2459	oxidoreductase	**-2.057287**
A1S_0445	hypothetical protein	**-2.053172**
A1S_0347	oxidoreductase	**-2.048254**
A1S_2468	hypothetical protein	**-2.042985**
A1S_2266	hypothetical protein	**-2.040855**
A1S_3175	bacterioferritin	**-2.037905**
A1S_0706	hydrolase	**-2.037448**
A1S_2274	aminoacyl-histidine dipeptidase	**-2.033091**
A1S_2628	electron transfer flavoprotein subunit beta	**-2.026734**
A1S_2763	aromatic amino acid APC transporter	**-2.026113**
A1S_1400	ABC transporter	**-2.025515**
A1S_1910	ATP-binding protease component	**-2.020792**
A1S_0087	short-chain dehydrogenase	**-2.019756**
A1S_1273	hypothetical protein	**-2.013049**
A1S_1395	hypothetical protein	**-2.012018**
A1S_2699	transcriptional regulator	**-2.009574**
A1S_2034	hypothetical protein	**-2.008047**
A1S_0470	methionine biosynthesis protein	**-2.006831**
A1S_3190	shikimate kinase	**-2.004224**
A1S_3032	TonB-like protein	**-2.003333**
A1S_0290	hypothetical protein	**-2.001855**
A1S_3304	two-component response regulator	**-2.001462**

Difference of >2-fold (q value < 0.05).

**Table 2s. Bacterial strains and plasmids used in this study.**		
**Bacteria or plasmids**	**Relevant characteristics**	**Reference of source**
**Bacteria**		
A. baumannii		
ATCC 17978 wild-type	Tri^R	Lab stock
ppGpp deficient strain	ΔA1S_0579, Tri^R	Jung et al (2020)
Complementary strain	A1S_0579, Tri^R	Jung et al (2020)
GFP expressing strain	pWH1266 harboring sfGFP gene insert (pMI66)	This study
**Plasmids**		
pMR-059(PT3-sfGFP)	sfGFP, T1 terminator, repA, BB prefix, amp^R	Lab stock
pWH1266	Shuttle-vector with *Acinetobacter* and *E. coli* origin used for cloning vehicle; amp^R; tet^R	Lab stock
pWH1266 harboring sfGFP gene insert (pMI66)	Amp^R<br>Tet^R	This study
Tri^R, trimethoprim resistant; amp^R, ampicillin resistant; tet^R, tetracycline resistant.		

Jung, H. W., Kim, K., Islam, M. M., Lee, J. C. & Shin, M. Role of ppGpp-regulated efflux genes in Acinetobacter baumannii. *J Antimicrob Chemother* **75**, 1130-1134, doi:10.1093/jac/dkaa014 (2020).

Table 3s. Oligonucleotides used in this study.

Primers	Sequence
GFP expressing strain.	
OmpAp F (Gibson assembly)	ACGTTGTTGCCATTGCTGCAGCGAGTGTTATAGTG AGCTCAACTGTA
OmpAp R	GGATATCCTCCAGAGATAACAATTGTTG
GFP F	TTATCTCTGGAGGATATCCATGCGTAAAGGCGAAGAGC
T1 terminator R (Gibson assembly)	ACTTCTGACAACGATCGTCTAGGGCGGCGGATTTG
pWH1266 F (Gibson assembly)	AATCCGCCGCCCTAGACGATCGTTGTCAGAAGTAAGTTGGC
pWH1266 R (Gibson assembly)	CTCACTATAACACTCGCTGCAGCAATGGCAACAAC
Primers used in real time PCR	
16S rRNA-sense	ACT CCT ACG GGA GGC AGC AGT
16S rRNA-antisense	TAT TAC CGC GGC TGC TGG C
CsuC-sense	AAAGCAGGCGAGAAGCATATG
CsuC-antisense	GGATCGGCAACTCATCTACAATC
CsuD-sense	ACCCTATCAAGGCGGTTCAAC
CsuD-antisense	CGACGATAGCCGTCATTATCTACA
CsuE-sense	TCAGACCGGAGAAAAACTTAACG
CsuE-antisense	GCCGGAAGCCGTATGTAGAA
BfmS-sense	TTGAACTTATTCCACCGCCTTT
BfmS-antisense	GCCCGTAATCCGAACTTTGTT
BfmR-sense	GTTTAACCGTTTGTCGTG
BfmR-antisense	GTGGTTGAACTGGTTTCG

A1S_1921-sense	TGTGTCTGCGTCTACATTTGTATCTAA
A1S_1921-antisense	AGACGAACTTGTGTGGAGTAAAGCT
A1S_2081-sense	CCGCAAGATGGTGCTGAAC
A1S_2081-antisense	CGGTTTGTCCAAGTTTCTGTTG
A1S_2389-sense	CGATCGGCACTTCAAATGG
A1S_2389-antisense	GCTAAAACCTTGCCGATTGTG
A1S_0414-sense	GGTCACAATGCTTGGCAATTT
A1S_0414-antisense	GCCGATTTCTTCTCTCAGTTCTC
A1S_2182-sense	GTCTCCGGTAATGTCGTTCAT
A1S_2182-antisense	GGTCTAAGCCACCACGAATAA
A1S_2813-sense	ACAGCGATGGTCAGGAATTG
A1S_2813-antisense	AACTGGTGTATATTTCGGTGGATAG
A1S_2814-sense	GCCAGATTACCCGTGATGAA
A1S_2814-antisense	GATTGCGCCTTGACGTTTAC
A1S_2815-sense	GAAACTCTATTGCAACGCGAAG
A1S_2815-antisense	AACGAGGCATCATGATATCTACAA
A1S_0049-sense	ACCGGCTCAAGCAGAAATAG
A1S_0049-antisense	TCACTCAATAAGCGATGGGTAAG
A1S_0050-sense	GCAGCGTTTAGGCATTGATATG
A1S_0050-antisense	GCAAAGGGCCAAGTTTGTT
A1S_0061-sense	AAAGGTGACATGAGCGTAGTG
A1S_0061-antisense	CATTCCTGGGCGAACTTCTAA
A1S_0062-sense	GCTCTGCACCATCAAACTTATC
A1S_0062-antisense	AATTTCATTACCCGCACCTTTC
